# Elevated GRO-α and IL-18 in serum and brain implicate the NLRP3 inflammasome in frontotemporal dementia

**DOI:** 10.1038/s41598-023-35945-4

**Published:** 2023-06-02

**Authors:** Hiu Chuen Lok, Jared S. Katzeff, John R. Hodges, Olivier Piguet, YuHong Fu, Glenda M. Halliday, Woojin Scott Kim

**Affiliations:** 1 Brain and Mind Centre, The University of Sydney, Camperdown, Sydney, NSW 2050 Australia; 2 School of Medical Sciences, The University of Sydney, Sydney, NSW Australia; 3 School of Psychology, The University of Sydney, Sydney, NSW Australia

**Keywords:** Cell biology, Neuroscience

## Abstract

Neuroinflammation is a hallmark of frontotemporal dementia (FTD), a heterogeneous group of proteinopathies characterized by the progressive degeneration of the frontal and temporal lobes. It is marked by microglial activation and subsequent cytokine release. Although cytokine levels in FTD brain and CSF have been examined, the number of cytokines measured in each study is limited and knowledge on cytokine concentrations in FTD serum is scarce. Here, we assessed 48 cytokines in FTD serum and brain. The aim was to determine common cytokine dysregulation pathways in serum and brain in FTD. Blood samples and brain tissue samples from the superior frontal cortex (SFC) were collected from individuals diagnosed with behavioral variant FTD (bvFTD) and healthy controls, and 48 cytokines were measured using a multiplex immunological assay. The data were evaluated by principal component factor analysis to determine the contribution from different components of the variance in the cohort. Levels of a number of cytokines were altered in serum and SFC in bvFTD compared to controls, with increases in GRO-α and IL-18 in both serum and SFC. These changes could be associated with NLRP3 inflammasome activation or the NFκB pathway, which activates NLRP3. The results suggest the possible importance of the NLRP3 inflammasome in FTD. An improved understanding of the role of inflammasomes in FTD could provide valuable insights into the pathogenesis, diagnosis and treatment of FTD.

## Introduction

Neuroinflammation is recognized as a hallmark for neurodegenerative diseases including frontotemporal dementia (FTD), a heterogeneous group of neurodegenerative clinical syndromes characterized by progressive behavioral and/or language changes and associated cognitive deficits^1^. There are three clinical subtypes of FTD: behavioral variant FTD (bvFTD), nonfluent variant primary progressive aphasia and semantic variant primary progressive aphasia, with bvFTD being the most common^2^. Neuropathologically, FTD is categorized based on the pathological cellular inclusions, with tau and Tar-DNA binding protein-43 (TDP-43) being the most prevalent, and both of which are known to trigger neuroinflammation^3,4^. Neuroinflammation is primarily mediated by activated astrocytes and microglia, the resident immune cells of the central nervous system. Microglia are mainly responsible for maintaining homeostasis and mediating host defense against pathogens and toxic protein aggregates. These stimuli trigger the activation of microglia, which releases pro-inflammatory cytokines, chemokines and reactive oxygen species (ROS). While early studies referred to four primary features of neuroinflammation—microglial activation, increased cytokines/chemokines, recruitment of peripheral immune cells and local tissue damage—the definition for this term has since broadened to cover most immune processes in the nervous system^5^. Physiologically, consequences of neuroinflammation include elevated ROS/oxidative stress, neuronal cell death, impaired phagocytosis and autophagy, mitochondrial dysfunction and protein aggregation^6–9^, all of which are known to contribute to the pathogenesis of neurodegenerative diseases^10–13^.

Neuroinflammation in FTD brain is evident by the presence of activated microglia in disease-affected regions, as shown by immunohistochemistry^14–18^. Indeed, the presence of activated microglia in the brain of FTD patients have been confirmed in vivo by positron emission topography (PET) using inflammation markers C-PK11195 or C-PBR28 that bind to the 18-kDa translocator protein (TPO) of activated microglia^16,19–22^. Furthermore, these studies showed a positive correlation between neuroinflammation and protein aggregations^16^ and disease progression^21^ across the FTD spectrum and in semantic dementia, respectively. Of note, PET imaging of seven cases of familial FTD with mutations in the three most common FTD causal genes, *C9ORF72*, *MAPT* and *GRN*, all showed in vivo inflammation, suggesting neuroinflammation is a part of the pathophysiology of familial FTD^22^. These three genes, which account for 35–65% of familial FTD cases^23^, and other FTD causal genes^24^ are all implicated in neuroinflammation^25–28^. Interestingly, the brain of *MAPT* mutation carriers show microglial activation in disease-affected regions prior to the development of protein aggregation and atrophy^28^, while the leukocytes from symptomatic *GRN* mutation carriers have increased expression of inflammatory genes compared to those of healthy controls^29^. In addition, the FTD risk gene *TREM2* has been reported to attenuate neuroinflammation^30,31^ and is used as a biomarker for microglial activation^32^.

Microglial activation leads to the release of cytokines that amplify and modulate the innate immune response to a foreign pathogen through binding to specific receptors and activating signaling cascade pathways to alter gene expression^33,34^. This allows cells to communicate with one another and orchestrate complex multicellular processes^35^. Alterations in brain and CSF cytokines levels in neurodegenerative diseases such as Alzheimer’s disease, Parkinson’s disease, amyotrophic lateral sclerosis and FTD have been reported^36–47^. Symptomatic FTD patients with *GRN* mutations have altered levels of pro-inflammatory cytokines in the serum and CSF^48,49^. Monocyte chemoattractant protein-1 (MCP-1), interferon-γ-inducible protein 10 (IP-10), IL-6, IL-11, IL-12 and IL-15, tumor necrosis factor β (TNFβ) and leukemia inhibitory factor (LIF)^46,49–53^ are all altered in the CSF or blood of FTD patients. Of note, the cytokines interleukins IL-1β and IL-18 are crucial for the NLRP3 inflammasome-mediated inflammatory responses^54^. NLRP3 inflammasomes are multi-protein complex comprised of a sensor (NLRP3), an adaptor (ASC) and an effector (caspase-1). The complex cleaves the pro-inflammatory interleukins IL-1β and IL-18, leading to their activation and release, resulting in a plethora of inflammatory responses including pyroptosis. NLRP3 inflammation activation have recently been implicated in driving the tau pathology^55^. The relationship between NLRP3 inflammasome and tau appears to be reciprocal. Activation of NLRP3 inflammasome can be caused by tau seeds in primary microglia^56^ in FTD brain with tauopathy^55^. Inflammasome activation is also known to regulate TDP-43 expression^57^. In addition, amyloid-β and α-synuclein were reported to induce NLRP3 inflammasome activation in Alzheimer’s^58,59^ and Parkinson’s^60^ disease, respectively.

Considering the importance of cytokines in neuroinflammation and neurodegenerative diseases, an improved understanding of their different roles in FTD could provide insights into the pathogenesis of FTD and other degenerative diseases. Currently, while there are numerous reports on cytokine changes in FTD, most of these analyses were performed on CSF, with little published examining cytokine changes in serum^24^ or in the brain, and only a small number of cytokines were measured. Furthermore, no direct comparisons were made between serum and brain cytokine levels; this would enable the identification of common cytokines that are altered in both the brain/CSF and serum in FTD. This is of particular significance as such cytokines could act as reliable neuroinflammation biomarkers that is detectable in the serum, thus providing a non-invasive means to assess neuroinflammation. In this current study, 48 cytokines were measured in the blood, using a multiplex assay, and were compared to those in the brain in bvFTD and healthy controls. The data were then evaluated by principal component factor analysis to understand the contribution from different components of the variance in our cohort. The primary aim was to determine whether cytokine dysregulation is evident in the serum and brain in FTD, and to identify common cytokines that are altered in both blood and the brain. The second aim was to examine whether any of these affected cytokines had similar functional roles that could lead to the discovery of hitherto unknown neuroinflammatory pathways that contribute to the pathogenesis of FTD.

## Materials and methods

### Participant blood serum

Individuals diagnosed with sporadic bvFTD and healthy controls were recruited from FRONTIER, the frontotemporal dementia clinical research group previously at Neuroscience Research Australia and now at the University of Sydney Brain and Mind Centre, and from a panel of healthy study volunteers^61^ with no neurological (i.e. no evidence of cognitive or motor impairment) or psychiatric disorders. The study was approved by the University of New South Wales human ethics committee (approval number: HC12573). All methods were carried out in accordance with the relevant guidelines and regulations. Blood samples were obtained following written informed consent from the participant and/or primary carer. All patients and controls underwent a neurological examination, a comprehensive cognitive assessment and structural brain MRI, and met current consensus diagnostic criteria for probable bvFTD^62^, as previously described^61^. Ten bvFTD cases (6 male, 4 female) and 10 controls (4 male, 6 female) were used in this study (Table 1). The mean age of the two groups at recruitment were 66.9 and 75.9 years, respectively. Two blood samples were collected from each person 12-months apart (i.e. Year-1 and Year-2), i.e. 40 samples in total. Blood samples (9 mL) were collected in tubes (BD Vacutainer SST II Advance Tube #367958), and serum prepared by centrifugation at 3500 rpm for 10 min at 4 ℃, which was then aliquoted and stored at −80 ℃ until use.

**Table 1:** Demographics of bvFTD and controls for serum analysis.

Case	Sex	Age at year 1	Age at year 2
bvFTD			
1	Male	79	80
2	Male	69	70
3	Female	73	74
4	Male	50	51
5	Male	64	65
6	Female	44	45
7	Female	67	68
8	Female	80	81
9	Male	61	62
10	Male	82	83
Control			
1	Male	80	81
2	Female	73	74
3	Male	75	76
4	Male	83	84
5	Female	80	81
6	Female	79	80
7	Female	76	77
8	Female	62	63
9	Male	76	77
10	Female	75	76

### Participant brain tissues

A different cohort of bvFTD patients and controls (Table 2) was used for the brain tissue analysis. Fresh-frozen post-mortem brain tissue samples were obtained with consent from the Sydney Brain Bank at Neuroscience Research Australia and NSW Brain Tissue Resource Centre at the University of Sydney (both brain banks ethically approved through their institutions to collect, characterize and bank brain tissue for research purposes). Ethics approval for this tissue study was from the University of New South Wales Human Research Ethics (approval number: HC15789). All brain donors underwent standardized assessments in life and standardized neuropathological examination, and met current consensus diagnostic criteria for sporadic bvFTD with TDP-43 pathology^63,64^ or no significant neuropathology (controls)^65,66^. The Sydney Brain Bank collects brain tissue from brain donors participating in the FRONTIER brain donor program approved through the South Eastern Sydney Local Health District Human Research Ethics (approval number: HREC 10/092) and so the bvFTD cases with brain tissue were clinically assessed via the same procedures as indicated for the patient blood serum. Tissue samples from the superior frontal cortex were collected from ten bvFTD cases (5 male, 5 female)^67^ and 11 controls (5 male, 6 female)^68^. The mean age of the two groups were 72.9 and 79.5 years, respectively. Pathological severity of FTD was also assessed within the bvFTD group which could be split further into early stage 1 (N = 5) versus later stage disease (N = 5)^69^, reflecting the average disease durations of these two subgroups (mean ± standard deviation for stage 1 disease duration of 2.7 ± 1.5 years versus stage 2/3 disease durations of 8 ± 4 years).

**Table 2:** Demographics of bvFTD and controls for brain tissue analysis.

Case	Sex	Age	PMI	Case characterization
FTD				
1	Male	66	39	Early FTD-TDP
2	Male	62	15	Early FTD-TDP
3	Female	72	25	Early FTD-TDP
4	Male	61	37	Early FTD-TDP
5	Female	65	22	Late FTD-TDP
6	Female	84	17	Late FTD-TDP
7	Male	60	28	Early FTD-TDP
8	Female	99	13	Late FTD-TDP
9	Female	86	25	Late FTD-TDP
10	Male	74	20	Late FTD-TDP
Control				
1	Female	85	23	N/A
2	Male	79	8	N/A
3	Female	89	23	N/A
4	Female	101	9	N/A
5	Male	84	9	N/A
6	Female	93	15	N/A
7	Male	74	10	N/A
8	Male	63	24	N/A
9	Male	66	23	N/A
10	Female	74	20	N/A
11	Female	67	15.5	N/A

### Protein fraction extraction from human brain tissues

The TBS fractions, which contain the cytosolic proteins, were used in cytokine analysis. The TBS fractions were extracted from the superior frontal cortex as previously described^68^. Briefly, tissue (100 mg) was suspended in TBS homogenization buffer (20 mM Tris, 150 mM NaCl, pH 7.4, 5 mM EDTA, 0.02% sodium azide) containing protease inhibitor cocktail (Roche). The samples were then homogenized using Qiagen tissue lyser (30 Hz cycles, 3 × 30 s) and centrifuged for 1 h (100,000×*g* at 4 °C). The resultant supernatant then becomes the TBS fraction. Samples were stored at −80 °C until analysis.

### Cytokine assay

A total of 48 cytokines including interleukins, chemokines, colony stimulating factors, growth factors, interferons, growth factors and tumor necrosis factor were measured in this study. Cytokine concentrations were measured in serum and human brain tissue TBS extracts using the Pro Human Cytokine Screening Panel 48-plex assay (Bio-Rad, Hercules, California, USA) and 5-point standard curve consisting of S3, S4, S5, S6, S7 standards and a blank, which covered our sample concentration range. Briefly, samples were diluted (serum 1:4, brain tissue 1:2), in sample diluent, and incubated with detection antibodies coupled to magnetic beads, washed using a Bio-Plex Pro wash station and incubated in streptavidin–phycoerythrin before wells were quantified using a Xponent software package (Luminex, Austin, TX). Provided standards generated a five-parameter standard curve for all 48 cytokines and unknown concentrations were calculated with Bio-Plex Manager software 6.1. The intra-assay %CV for the serum plate was 2.68–4.97 (average: 3.51) and for the brain tissue plate 3.65–6.42 (average: 4.86). The inter-assay %CV for the two plates was 4.48–13.57 (average: 7.92).

### Statistical analysis

All statistical analyses were performed using SPSS statistical software (IBM, Chicago, IL, United States). A multivariate analysis (general linear model), covarying for age and sex, was used to determine differences in the cytokine levels in FTD (N = 10) and control (N = 11) with posthoc statistical significance set at P < 0.05. Principal component factor analyses (PCA) were performed to determine if significantly altered cytokines, were clustering in the same group of variance for serum and brain cytokines. To be considered significant, cytokines required a loading score of > 0.7 and to be responsible for > 10% of variance. PCA was first performed on serum cytokines to determine if cytokines that were altered in the serum of bvFTD cohort were clustering together. This is followed by analysis on brain cytokines to examine if similar components of variance were observed in both brain and serum.

## Results

### Altered cytokine levels in FTD serum

**Figure 1:**
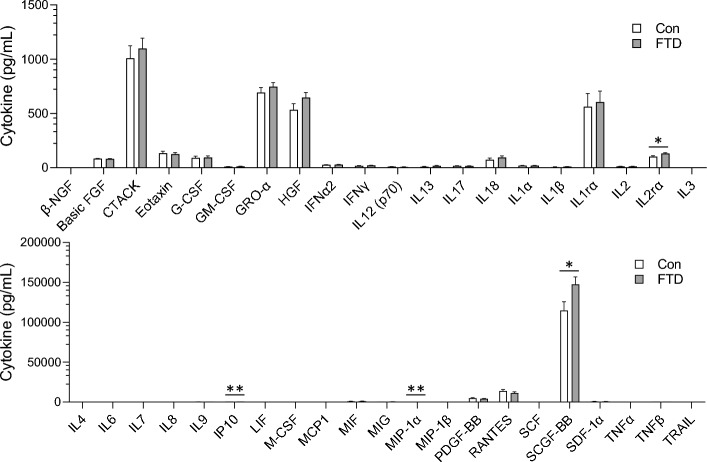
Serum cytokines levels in bvFTD and control cases. Serum samples collected from year 1 and year 2 of FTD (20 samples) and control cases (20 samples) were quantified for cytokine levels and the results were grouped according to disease state. White bars show control and light grey are bvFTD. Data represent mean and SE as error bars, *P < 0.05, **P < 0.01, ***P < 0.001.

**Figure 2:**
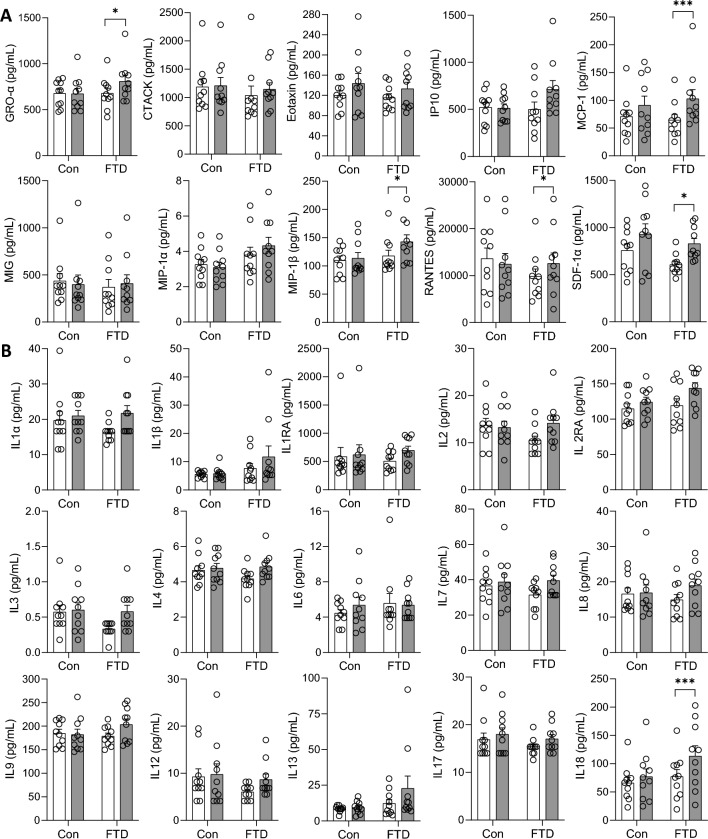
Serum chemokines and interleukins levels in year 1 and year 2 of bvFTD and control cases. Serum samples collected from year 1 and year 2 of FTD (20 samples) and control cases (20 samples) were quantified and the levels of from each year were grouped for control and bvFTD cases. The results for **(A)** chemokines and **(B)** Interleukins were shown here. White bars show results from year 1 and light grey are results from year 2. Data represent mean and SE as error bars, *P < 0.05, **P < 0.01, ***P < 0.001.

**Figure 3:**
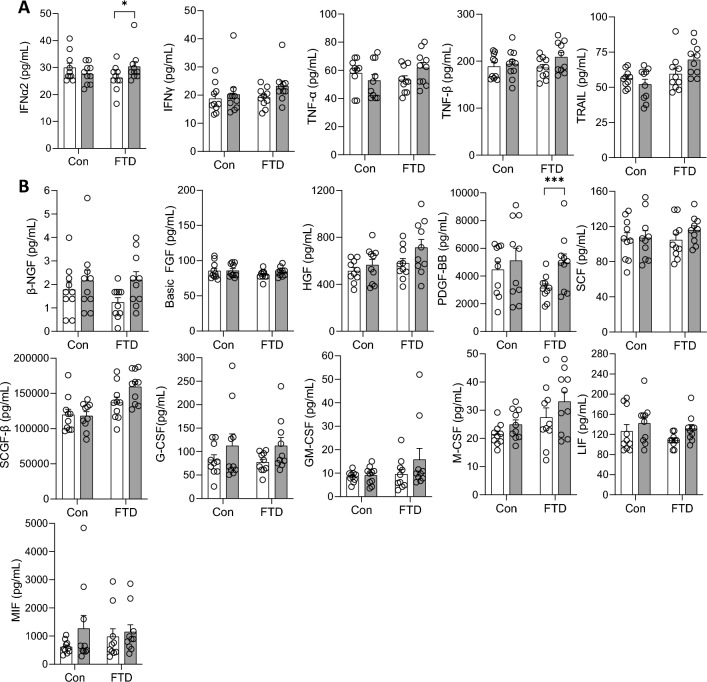
Serum interferons, growth factors and colony stimulating factors levels in year 1 and year 2 of bvFTD and control cases. Serum samples collected from year 1 and year 2 of FTD (20 samples) and control cases (20 samples) were quantified and the levels of from each year were grouped for control and bvFTD cases. The results for **(A)** interferons and **(B)** growth factors and colony stimulating factors were shown here. White bars show results from year 1 and light grey are results from year 2. Data represent mean and SE as error bars, *P < 0.05, **P < 0.01, ***P < 0.001.

Forty-eight cytokines were measured in bvFTD (N = 10) serum and controls (N = 10) using a multiplex assay. Two samples, year 1 and year 2 (i.e. 12-months apart), from each individual were assessed. Firstly, we assessed the cytokines independent of time and found that IL-2Rα, IP10, macrophage inflammatory protein 1-alpha (MIP-1α) and stem cells growth factor-beta (SCGF-BB) were significantly increased in bvFTD compared to controls (Fig. 1). Of the 48 cytokines, IL-10, IL-12 (p40), IL-5, IL-15, IL-16, monocyte chemotactic protein-3 (MCP-3) and vascular endothelial growth factor (VEGF) were not detected by the multiplex assay. Secondly, we assessed the cytokines longitudinally and found that five chemokines (GRO-α/CXCL1, monocyte chemotactic protein-1 (MCP-1), macrophage inflammation protein 1-beta (MIP-1β), RANTES and SDF-1α) (Fig. 2A), one interleukin (IL-18) (Fig. 2B), one interferon (interferon alpha-2 (IFNα-2)) (Fig. 3A) and one growth factor (platelet derived growth factor BB (PDGF-BB)) (Fig. 3B) were significantly altered in year 2 compared to year 1 in bvFTD. None of the cytokines were altered in year 2 compared to year 1 in controls (Figs. 2 and 3).

### Cytokine analysis of FTD brain tissue

**Figure 4:**
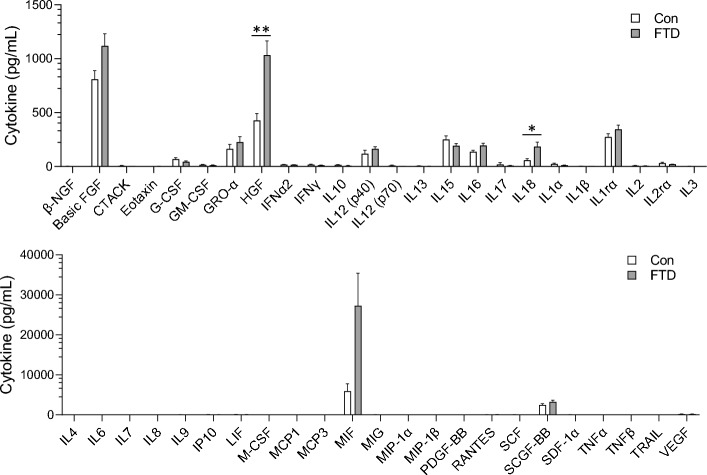
Brain cytokines levels in bvFTD and control cases. Cytokine levels in the frontal cortex of FTD (10 samples) and control cases (10 samples) were quantified and the results were grouped according to disease state. White bars show control and light grey are bvFTD. Data represent mean and SE as error bars, *P < 0.05, **P < 0.01, ***P < 0.001.

**Figure 5:**
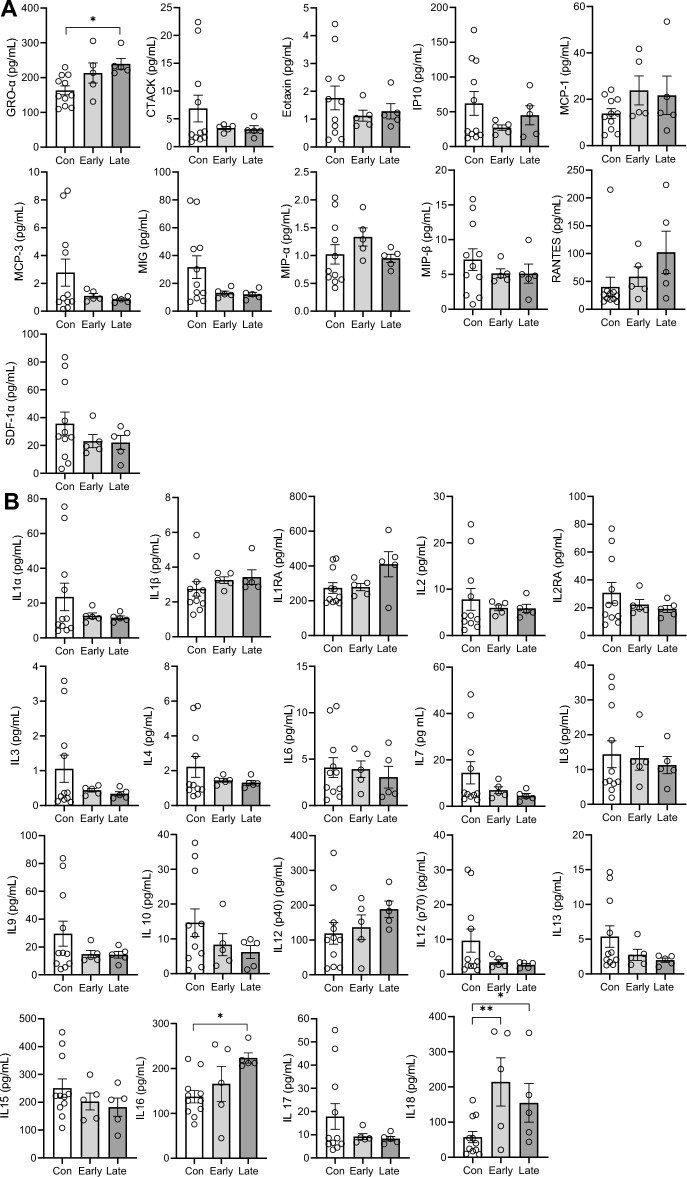
Chemokines and interleukins levels in early and late bvFTD brains compared to control cases. Cytokine levels in the frontal cortex of FTD (10 samples) and control cases (10 samples) were quantified and the bvFTD cases were further separated into early cases (5 cases) and late cases (5 cases). The results for **(A)** chemokines and **(B)** Interleukins were shown here. White bars show results from year 1 and light grey are results from year 2. Data represent mean and SE as error bars, *P < 0.05, **P < 0.01, ***P < 0.001.

**Figure 6:**
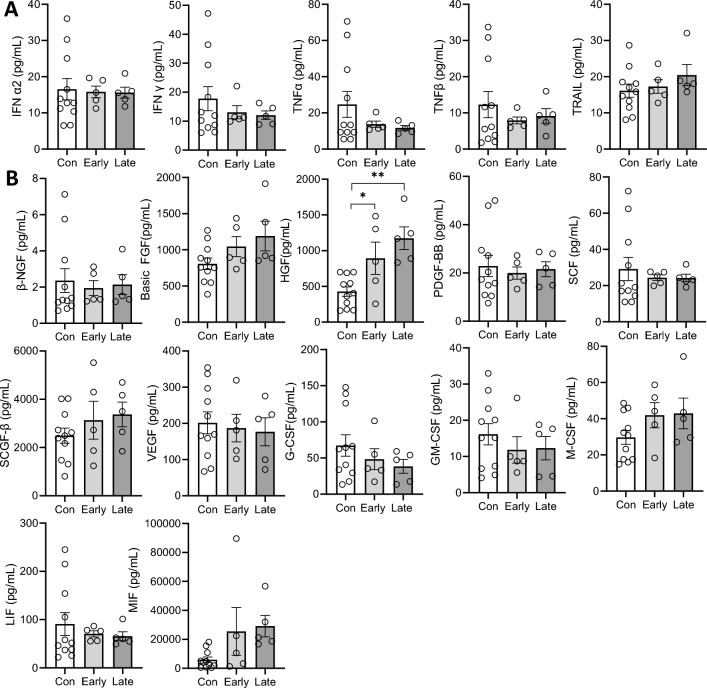
Interferons, growth factors and colony stimulating factors levels in early and late bvFTD brains compared to control cases. Cytokine levels in the frontal cortex of FTD (10 samples) and control cases (10 samples) were quantified and the bvFTD cases were further separated into early cases (5 cases) and late cases (5 cases). The results for **(A)** interferons and **(B)** growth factors and colony stimulating factors were shown here. White bars show results from year 1 and light grey are results from year 2. Data represent mean and SE as error bars, *P < 0.05, **P < 0.01, ***P < 0.001.

We were also interested in changes in the cytokines in bvFTD brain and assessed the same cytokines, using the same multiplex assay, in the superior frontal cortex, a disease-affected region, of FTD (N = 10) and controls (N = 11). Of the 48 cytokines, HGF and IL-18 were significantly elevated in bvFTD compared to controls (Fig. 4) with IL-5 being undetectable. We then categorized bvFTD into two groups based on neuropathological severity, i.e. early stage 1 bvFTD (N = 5) and later stage 2/3 bvFTD (N = 5), and divided the cytokines into functional groups, i.e. chemokines, interleukins, interferons, growth factors and colony stimulating factors. In terms of chemokines and interleukins, GRO-α (Fig. 5A) and IL-16 (Fig. 5B) were significantly elevated in late bvFTD compared to controls, whereas IL-18 was more significantly increased in early bvFTD relative to late bvFTD (Fig. 5B). In terms of interferons, there were no changes in either early or late bvFTD compared to controls (Fig. 6A). In terms of growth factors, HGF was increased in early bvFTD and further increased in late bvFTD compared to controls (Fig. 6B). In summary, GRO-α and IL-18 are elevated in both serum and brain in bvFTD compared to controls.

### Analysis of variance in brain and serum cytokines

**Figure 7:**
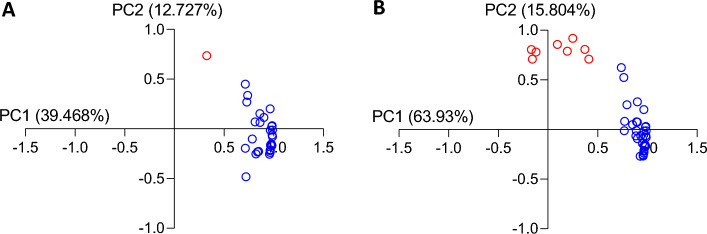
Principal components Analysis (PCA) on significantly altered cytokines in **(A)** serum and **(B)** brain. PCA were performed to determine whether cytokines with significantly altered levels in FTD samples were clustering in the same group of variance. To be considered significant, cytokines required a loading score of > 0.7 and to be responsible for > 10% of variance. PCA on (**A**) serum and (**B**) brain datasets both reveal two components of variance. Cytokines in component 1 are represented by blue circles while cytokines in component 2 are represented by red circles (component 2).

To identify clustering groups of cytokines with similar functional characteristics in bvFTD serum and brain (> 10% variance from controls), principal factor component analysis (PCA) was performed on the serum (Fig. 7A) and brain (Fig. 7B) datasets. The serum PCA revealed two components of variance. The larger component (loading score > 0.7, accounting for 39% of variance) comprised of beta-nerve growth factor (β-NGF), granulocyte colony stimulating factor (G-CSF), IFN-α2, IL-17A, IL-1α, IL-2, IL-3, IL-4, IL-7, IL-8, IL-9, MIP-1β and tumor necrosis factor-alpha (TNF-α) (Table 3). The other component consisted of only one cytokine, RANTES.

**Table 3:** Cytokines related to largest component of variance in bvFTD and control serum.

Cytokine	Loading score
Group 1	
β-NGF	0.901
TNF-α	0.863
G-CSF	0.860
IL-1α	0.848
IL-2	0.837
IL-4	0.820
IL-8	0.813
IL-3	0.784
MIP-1β	0.737
IL-9	0.729
IFN-α2	0.722
IL-17A	0.715
IL-7	0.714
Group 2	
RANTES	0.737

To determine whether similar components of variance occurred in serum and brain, PCA analysis was then performed on all brain samples, from which two components of variance emerged (Table 4). The analysis showed that the greatest component of variance in FTD brain consisted of β -NGF, eotaxin, G-CSF, granulocyte–macrophage colony stimulating factor (GM-CSF), IFN-α2, IFN-γ, IL-10, IL-12 (p40), IL-12 (p70), IL-13, IL-15, IL-16, IL-17A, IL-1α, IL-2Rα, IL-3, IL-4, IL-6, IL-7, IL-8, IL-9, LIF, MCP-3, monocyte induced by gamma interferon (MIG/ CXCL9), MIP-1α, MIP-1β, PDGF-BB, stem cell factor (SCF), stromal cell derived factor-1 alpha (SDF1α), TNF-α, TNF-β and VEGF (loading score > 0.7, accounting for 51% of variance) (Table 4). The second largest component of variance consisted of fibroblast growth factor (FGF basic), GRO-α, hepatocyte growth factor (HGF), IL-16, IL-18, M-CSF, macrophage migration inhibitory factor (MIF) and SCGF-β (loading score > 0.7, accounting for 15% of variance). Interestingly, the second largest component of variance comprised of all four cytokines that were altered in the brain—GRO-α, HGF, IL-16 and IL-18. In addition, all the cytokines from the first components of variance in the serum samples were also found in the largest component of variance in the brain.

**Table 4:** Cytokines related to largest components of variance in superior frontal cortex.

Cytokine	Loading score
Group 1	
IL-2Rα	0.988
LIF	0.987
IFN-γ	0.984
SCF	0.983
TNF-α	0.982
IL-2	0.981
IL-9	0.981
IL-17A	0.979
IL-4	0.976
IL-1α	0.974
TNF-β	0.966
IL-3	0.965
IL-7	0.965
IFN-α2	0.965
IL-13	0.964
MCP-3	0.957
IL-12 (p70)	0.957
SDF-1α	0.952
IL-10	0.950
G-CSF	0.932
MIG	0.927
IL-8	0.899
MIP-1β	0.899
PDGF-BB	0.898
β-NGF	0.891
IL-15	0.887
Eotaxin	0.884
IL-6	0.851
VEGF	0.795
GM-CSF	0.772
MIP-1α	0.768
IL-1β	0.763
IL-12 (p40)	0.737
Group 2	
GRO-α	0.919
HGF	0.858
M-CSF	0.808
MIF	0.806
IL-18	0.790
FGF basic	0.781
IL-16	0.709
SCGF-β	0.709

## Discussion

Neuroinflammation is known to play a major role in the neuropathology of neurodegenerative diseases including bvFTD^24^. Since cytokines are integral to neuroinflammation, an improved understanding of cytokines in bvFTD could provide valuable insights into the pathogenesis of bvFTD and other neurodegenerative diseases. While changes in brain/CSF cytokines levels in bvFTD have been well documented, reports on serum cytokine concentrations in bvFTD are scarce, and the number of cytokines measured in these studies small. The present study is the first to directly compare the serum and brain levels of a comprehensive range of cytokines in bvFTD. The side-by-side assessment of serum and brain cytokine concentrations allowed the identification of common cytokines that are altered in both the blood and brain. The relative ease of serum collection over that of CSF makes these cytokines good candidates for neuroinflammation biomarkers. In addition, the grouping of functionally similar cytokines enabled the identification of novel neuroinflammation pathways that could contribute to the pathogenesis of bvFTD.

In agreement with previous studies, this study also showed cytokine level changes in serum and brain of bvFTD patients compared to those of healthy controls. In the serum, the concentrations of four cytokines, IL-2Rα, IP-10, MIP-1α and SCGF-BB, were elevated in bvFTD compared to controls, while GRO-α, IFN-α2, IL-18, MCP-1, MIP-1β and PDGF-BB have shown time-dependent increase in serum concentration with disease progression. Of note, GRO-α and IL-18 levels were also increased in bvFTD brain, in addition to HGF and IL-16. The fact that both bvFTD brain and serum have elevated levels of GRO-α and IL-18 suggests that these two cytokines are involved in pathways crucial in the pathogenesis of bvFTD. Principle component analysis on brain cytokines levels have placed GRO-α and IL-18 in the group 2 of brain cytokines (Table 4), suggesting that these two cytokines are functionally related in bvFTD brain. IL-18 and GRO-α (also known as CXCL1) are both pro-inflammatory cytokines. IL-18 is involved in the activation of mast cells and CD8 + T cells, production of IFN-γ and Th2 cytokines and inducing innate-type allergic inflammation^70^ while GRO-α binds to its receptor CXCR2 to promote neutrophil recruitment and activation at the site of infection. In addition, both cytokines are involved in NLRP3 inflammasome pathways. While IL-18 release is mediated by the NLRP3 inflammasome activation, which recruits caspases-1 to cleave IL-18 pro-peptide to active IL-18^71,72^, GRO-α has been shown to promote the activation of NLRP3 inflammasome^73^. Although dysregulation of these two cytokines were unknown in bvFTD, an increase in IL-18^74,75^ and GRO-α^76^ have been reported in other neurodegenerative diseases^77,78^.

Apart from IL-18 and GRO-α, the second components of variance for brain cytokines (Group 2) also contained HGF, M-CSF, MIF, FGF basic, IL-16 and SCGF-β. Of note, GRO-α, HGF, IL-16 and IL-18 levels were all altered (Fig. 3), which is suggestive of their significant physiological roles in bvFTD brain. Apart from SCGF-β—a recently discovered protein for which very little is known–the other cytokines in the clusters are involved in the NLRP3 inflammasome pathway. FGF has been shown to upregulate the NLRP3 inflammasome^79^, while MIF is required for NLRP3 activation^80^. Interestingly, HGF is known to inhibit the NFκB pathway^81^ leading to non-expression of RANTES, MCP-1, IL-1β, TNF-α, IL-1 and IL-6^81^. Significantly, the NFκB pathway is known to activate the NLRP3 inflammasome^82^. On the other hand, M-CSF have been shown to activate NFκB^83^. Thus, the cytokines clustered in group 2 of brain cytokines are involved in the NLRP3 inflammasome pathway, either directly or through the NFκB pathway, thus underscoring the importance of NLRP3 inflammasome in the etiology of bvFTD.

The significance of the NFκB pathway in the pathogenesis of FTD was further confirmed by results emerged from the principal component analyses, in which cytokines from the first component of variance for brain cytokines, group 1 (Table 4), are all related to the NFκB signaling pathway, either as NFκB regulators and/or downstream effectors of the NFκB pathway. IL-2R α, IFNγ, TNFα, IL-2, IL-17A, IL-4, IL-1α, TNF-β, IFN-α2, IL-13, IL-12 (p70), SDF-1α, G-CSF, PDGF-BB, β-NGF, IL-15, IL-1β are all activators of NFκB^84–102^, while IL-9, IL-10 and IL-13 are reported to suppress NFκB^103–105^. In turn, some of these cytokines are activated by NFκB: TNF-α, IL-3, IL-8, MIP-1β, eotaxin, IL-6 and GM-CSF, MIP-1α^106–111^. In addition, LIF, SCF, IL-7 MCP-3, MIG, VEGF and IL-12 (p40) are regulated by the NFκB pathway^112–117^. Moreover, several of these cytokines also have a direct link to the NLRP3 inflammasome: the secretion of IL-1α and β are mediated by the NLRP3 inflammasome^54^, while IL-4 is reported to inhibit inflammasome assembly^118^ and SDF-1α inhibits inflammasome activation^119,120^. Notably, cytokines from the entire first components of variance for serum (Group 1), β-NGF, TNFα, G-CSF, IL-1α, IL-2, IL-4, IL-8, IL-3, MIP-1β, IL-9, IL-17A, and IL-7, are all present in the first component of variance for brain cytokines, again confirming the prominence of the NFκB pathway in the etiology of bvFTD. The second component for serum cytokines only consisted of one cytokine, RANTES, and it is also regulated by NFκB^121^. Of note, some of the above cytokines are also responsible for regulation of other cytokines. For example, IL-15 induces IL-8 production^100^, SDF1-α upregulates IL-6^122^ and GM-CSF signaling increases IL-1 production^123^, implying secondary regulatory mechanisms within these cytokines. Thus, the current study showed that significantly altered cytokines in bvFTD are all part of an intricate network that revolves around the NLRP3 inflammasome, either directly or via the NFκB pathway.

Activation of the NLRP3 inflammasome has been associated with neurodegenerative diseases^124–126^, including FTD^55^. Indeed, amyloid-β and α-synuclein were reported to induce NLRP3 inflammasome activation in Alzheimer’s^58,59,127^ and Parkinson’s^60^ disease, respectively. In addition, mutant SOD1 and TDP-43 proteins have also been reported to activate NLRP3 inflammasome^128^ while aggregated tau^56^ and TDP-43^57,129^ are also known to activate NLRP3 inflammasome. Interestingly, a recent study implicated NLRP3 inflammasome activation in driving tau pathology^55^. Indeed, inflammasome inhibitors have been shown to inhibit α-synuclein pathology^130^ and reduce amyloid-β accumulation^131^ in mouse models. Unsurprisingly, there is increasing interest in using NLRP3 inflammasome inhibitors as a therapeutic target for neurodegenerative diseases^132–134^. Pilot studies using inhibitors of NLRP3 in mouse models of neurodegenerative diseases have proved this approach effective^130,131,135–138^. In an FTD mouse model, the inflammasome inhibitor MCC950 improves inflammation and endoplasmic reticular stress signaling, in addition to partially normalizing the levels of phosphorylated tau^139^.

Taken together, the current study has revealed evidence of cytokine dysregulation in bvFTD serum and brain. In particular, the levels of IL-18 and GRO-α appear to be changed in both serum and brain of bvFTD patients, making these two cytokines possible inflammation biomarkers for bvFTD. Interestingly, these two cytokines are both involved in the NLRP3 inflammasome pathway, which has been associated with other neurodegenerative diseases. Furthermore, principal component analysis performed on serum and brain cytokines have revealed that all significantly altered cytokines are associated with the NLRP3 inflammasome and/or the NFκB pathway, which is a known activator for the NLRP3 inflammasome. Thus, our data show that NLRP3 inflammasome signaling occurs early in the pathogenesis of bvFTD. Given the recent interest in using NLRP3 inflammasome inhibitors as therapeutics against neurodegenerative diseases, and the promising outcomes of these molecules in mouse models, a better understanding on the role of cytokines in NLRP3 inflammasome activation could provide valuable insights into the pathogenesis of bvFTD and its diagnosis and treatment.

## Conclusions

In conclusion, our results showed that cytokine dysregulation is evident in bvFTD brain and serum. Importantly, GRO-α and IL-18 appear to be increased in both serum and brain in bvFTD, making them possible candidates as neuroinflammation biomarkers for bvFTD. The cytokines that are altered in bvFTD serum and/or brain are all related to NLRP3 inflammasome activation or NFκB pathway, which regulates NLRP3. These results therefore suggest that the NLRP3 inflammasome could be important in bvFTD pathogenesis.

## Data Availability

All relevant data are available from the corresponding author upon reasonable request. Other patient data cannot be made publicly available because the ethical approval and the informed consent from the patients included in this study did not cover placing the data into publicly open repositories.
